# Impact of Emergency Department Phlebotomists on Left-Before-Treatment-Completion Rates

**DOI:** 10.5811/westjem.2019.5.41736

**Published:** 2019-07-02

**Authors:** Jeffrey R. Stowell, Paul Pugsley, Heather Jordan, Murtaza Akhter

**Affiliations:** * University of Arizona College of Medicine-Phoenix, Department of Emergency Medicine, Phoenix, Arizona; † Maricopa Integrated Health System, Department of Emergency Medicine, Phoenix, Arizona; ‡ Creighton University School of Medicine, Department of Emergency Medicine, Omaha, Nebraska

## Abstract

**Introduction:**

The emergency department (ED) serves as the primary access point to the healthcare system. ED throughput efficiency is critical. The percentage of patients who leave before treatment completion (LBTC) is an important marker of department efficiency. Our study aimed to assess the impact of an ED phlebotomist, dedicated to obtaining blood specimen collection on waiting patients, on LBTC rates.

**Methods:**

This study was conducted as a retrospective observational analysis over approximately 18 months (October 5, 2015–March 31, 2017) for patients evaluated by a triage provider with a door-to-room (DtR) time of > 20 minutes (min). LBTC rates were compared in 10-min DtR increments for when the ED phlebotomist collected the patient’s specimen vs not.

**Results:**

Of 71,942 patient encounters occurring during the study period, 17,349 (24.1%) met study inclusion criteria. Of these, 1842 (10.6%) had blood specimen collection performed by ED phlebotomy. The overall LBTC rate for encounters included in the analysis was 5.26% (95% confidence interval [CI], 4.94%–5.60%). Weighting the LBTC rates for each 10-min DtR interval using the fixed effects model led to an overall LBTC rate of 2.74% (95% CI, 2.09%–3.59%) for patient encounters with ED phlebotomist collection vs 5.31% (95% CI, 4.97%–5.67%) in those which did not, yielding a relative reduction of 48% (95% CI, 34%–63%). The effect of the phlebotomist on LBTC rates increased as DtR times increased. The difference in the rate of the rise of LBTC percentages, per 10-min interval, was 0.50% (95% CI, 0.19%–0.81%) higher for non-ED phlebotomist encounters vs phlebotomist encounters.

**Conclusion:**

ED phlebotomy demonstrated a significant reduction in ED LBTC rates. Further, as DtR times increased, the impact of ED phlebotomy became increasingly significant. Adult EDs with increased rates of LBTC patient encounters may want to consider the implementation of ED phlebotomy.

## INTRODUCTION

The emergency department (ED) serves as the primary access point to the healthcare system for more than 117 million patient encounters in the United States (U.S.) annually.^1^ Prolonged wait times, extended lengths of stay (LOS), and crowding negatively impact the patient experience and quality of care.^2^,^3^ To meet patient and community healthcare needs, efficient ED throughput and patient flow is critical. Patients who leave the ED prior to completing assessment, treatment, and formal disposition by an ED provider have been identified as a potential marker of systemwide inefficiency.^4^

Patients who leave the ED before treatment completion (LBTC) represent the total number of patients who leave early.^5^ Overall, approximately 0.36%–15% of all patients presenting to an ED in the U.S. LBTC.^5^–^8^ Of these, approximately two-thirds leave before being seen (LBBS) by a physician or physician extender, with the remaining one-third leaving subsequent to being seen (LSBS).^6^ LBTC encounters increase ED recidivism, potentially damage the reputation and trust of the healthcare institution with the community, and result in lost revenue.^6^,^9^,^10^–^13^ These encounters are considered “missed opportunities” for the healthcare system.^9^,^14^ Accordingly, the proportion of LBBS encounters is used by the Centers for Medicare & Medicaid Services (CMS) as a hospital quality indicator, with previous investigators estimating the desirable LBBS goal at <2%.^4^,^15^

Excessive wait time, due to crowding and fluctuating patient volumes beyond ED capacity, is the most powerful LBTC predictor.^9^,^10^,^12^,^15^–^18^ The mean time a patient spends in the ED before they leave without being seen is between 102.4–171 minutes (min).^9^,^19^ Initiatives aimed at reducing ED LBTC rates commonly target the patient arrival process in order to reduce the time from patient arrival to room and formal evaluation.^12^ A target wait time of fewer than 45 mins, for patients who do not require the most immediate intervention or evaluation as characterized by an Emergency Severity Index (ESI) 3, and 60 mins for ESI 4 patients, has been demonstrated to result in an overall LBBS rate of < 2%.^15^ Further, a door-to-room (DtR) time of <20 mins increases the likelihood of obtaining a LBBS rates of <1%.^20^

We predict early patient engagement in a meaningful and tangible way increases the patient’s investment in the encounter and will therefore make them less likely to leave early. Our study aimed to describe the impact of blood specimen collection performed by a dedicated ED phlebotomist on patients waiting to be roomed, on LBTC rates as DtR times increase.

## METHODS

The study protocol was reviewed and approved by the Maricopa Integrated Health System institutional review board. The study did not involve human subjects.

### Study Setting and Population

The study ED is a large, urban, single-center, adult Level 1 trauma center at a primary academic training institution and is part of a safety net healthcare system. The annual ED census includes approximately 50,000 patient encounters, with a 14% admission rate and approximately 2000 hours of ED boarding of admitted patients per month. The ED is staffed by emergency medicine (EM)-boarded physicians, advanced practice providers (APPs), and EM residents in a postgraduate year (PGY) 1–3 program.

Upon arrival, patients are triaged by a registered nursing provider before moving to a bedded location in fast track (five beds), the main ED (32 beds), or a designated critical care area (five beds). Patients arriving via emergency medical services (EMS) are offloaded to a hallway bed before being moved to a room. For 12 hours a day, the triage encounter also includes a brief physician-in-triage screening assessment. Stable patients, unable to be roomed immediately due to ED saturation, wait in the ED external waiting room after triage is completed. The ED employs a single ED technician as a phlebotomist eight hours per day (1 pm–9 pm), four days a week (Monday, Tuesday, Thursday, Friday), in overlap with the physician-in-triage. The ED phlebotomist is tasked with blood specimen collection on orders placed during the triage process for patients waiting in the external waiting room prior to being roomed. For encounters occurring when ED phlebotomy is not available, a nurse collects a blood specimen collection after the patient has been roomed.

**Population Health Research Capsule**

What do we already know about this issue?

Increased emergency department (ED) wait times, lengths of stay, and patients who leave prior to completing treatment (LBTC) are a potential marker of systemwide inefficiency.

What was the research question?

Do dedicated ED phlebotomists decrease LBTC rates on patients waiting to being roomed as door-to-room (DtR) times increase?

What was the major finding of the study?

The LBTC rate for encounters with ED phlebotomy was 2.74% vs 5.31% in those without. The effect increased as DtR times increased.

How does this improve population health?

ED phlebotomy reduced LBTC rates as DtR times increased. EDs should consider the implementation of ED phlebotomy to reduce LBTC rates.

### Study Protocol

This study was conducted as a retrospective observational analysis over approximately 18 months (October 5, 2015–May 31, 2017). We extracted the following from the ED electronic health record for all patient encounters that occurred during the study window: 1) patient demographics, including gender, age and ESI; 2) whether blood specimen collection was ordered and performed by ED phlebotomy or nursing; 3) encounter throughput metrics, including arrival time to triage, room, blood specimen collection, provider and disposition; and 4) whether the patient completed treatment. Data extraction was performed by a blinded programmer and then reviewed by study authors who were not blinded to the study hypothesis.

LBTC rates for patient encounters when the ED phlebotomist collected the patient’s blood specimen were compared to encounters with nursing collection after the patient was roomed or when the patient did not require collection. We included for analysis only patient encounters with a screening evaluation by a triage physician and a DtR time of >20 mins for analysis. Encounters with undocumented or DtR time of <20 mins were excluded as previous studies have demonstrated very few patients roomed within 20 mins of arrival leave early.^20^ We also excluded encounters without a physician-in-triage screening encounter as ED phlebotomy was only available when the physician-in-triage was present, and it has previously been shown that a physician-in-triage screening encounter increases the number of patients who are willing to complete treatment.^21^ For analysis, encounters were stratified into 10-min DtR time increments (starting with DtR of >20–≤30 mins). Patient encounters with DtR times beyond six hours were grouped into a single stratum for analysis. Patient encounters without blood specimen collection orders were included, as the patient was unlikely to be aware of whether collection orders were placed when deciding to leave early.

### Data Analysis

Proportions are described with confidence intervals (CI) using Wilson method with continuity correction. To determine the overall percentage change in LBTC rates, we used a fixed effects model and calculated CIs using the law of propagation of uncertainty (and confirmed them using Monte Carlo simulation of the binomial distribution). Linear regression was performed to evaluate and compare the rate of LBTC increase as DtR times rose. We evaluated significance of the rate increase trend using Cochrane Armitage test. Statistical analysis was performed in Microsoft Excel (Microsoft Corp., Redmond, WA) and R version 3.5.1.

## RESULTS

**Figure 1:**
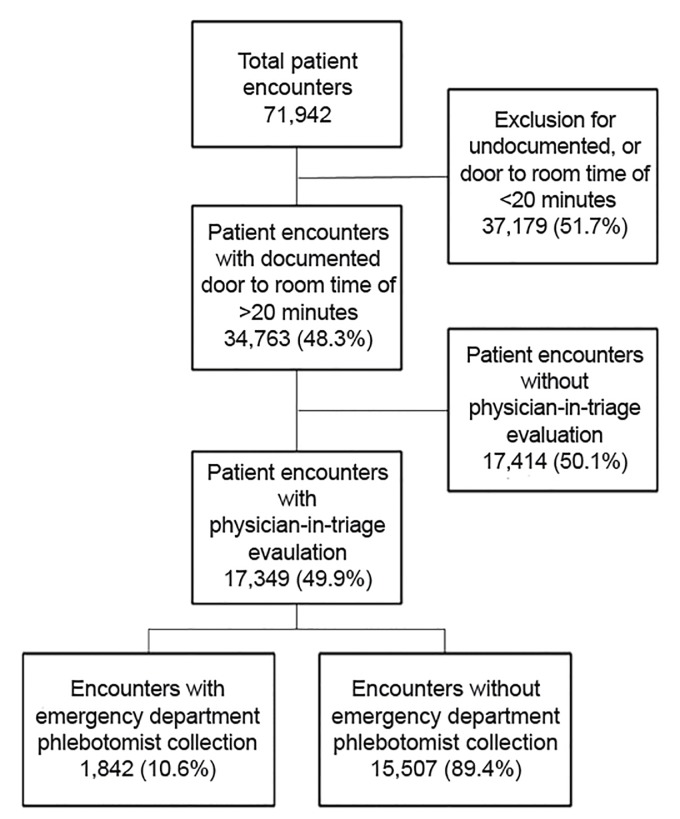
Study population inclusion for comparison of throughput with and without ED phlebotomy.

A total of 71,942 patient encounters occurred during the study period, of which 17,349 (24.1%) met study inclusion criteria (Figure 1). Additionally, one encounter was removed prior to analysis due to incomplete data. Of these, 1842 (10.6%) had blood specimen collection performed by ED phlebotomy prior to being roomed. Patient encounter demographics and throughput characteristics are described in Table 1. Encounters with ED phlebotomist collection were found to have a similar ESI (ESI 3), with an overall lower rate of admission (10.7%) as compared to those without (14.1%). The ED phlebotomist encounter group was found to have similar door to triage (17.98 mins vs. 18.48 mins), shorter door to blood specimen collection (66.16 mins v. 152.26 mins), and longer DtR (122.71 mins vs 74.12 mins), primary physician evaluation (207.10 mins vs 149.21 mins), and disposition times (343.76 mins vs 286.76 mins) as compared to the non-ED phlebotomist encounter group.

The overall LBTC rate for encounters included in the analysis was 5.26% (95% CI, 4.94%–5.60%). Weighting the LBTC rates for each ten-min DtR interval using the fixed effects model demonstrated an overall LBTC rate of 2.74% (95% CI, 2.09%–3.59%) for patient encounters with ED phlebotomist collection vs 5.31% (95% CI, 4.97%–5.67%) in those that did not, yielding a relative reduction of 48% (95% CI, 34%–63%). For encounters with DtR of <20 mins, which were excluded from the primary analysis, we found a significant difference in the LBTC rate for encounters with blood specimen collection performed by ED phlebotomy, as compared to ED nursing collection (1.68% vs. 2.57%, p < .001).

**Figure 2:**
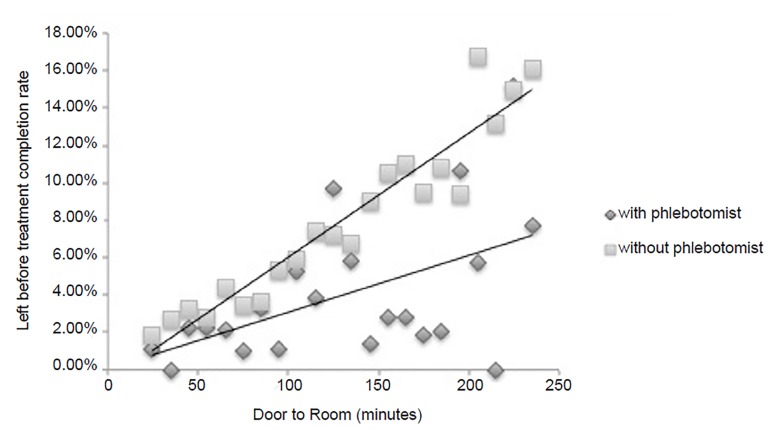
Left-before-treatment-completion percentage between time intervals 20 and 240 miutes.

Figure 2 demonstrates the LBTC rate at each 10-min interval between patients who had ED phlebotomist-collected specimens as compared to those who did not between DtR times of 20 mins to 240 mins. The effect of the phlebotomist on the LBTC rate increased as DtR times increased. (Cochrane Armitage test for trend was p < 0.01.) The difference in the rate of the rise of the LBTC percentage, per ten-min interval, was 0.50% (95% CI, 0.19%–0.81%) higher for non-ED phlebotomist encounters vs phlebotomist encounters. For encounters with DtR of >240 mins, which were excluded from the primary analysis, the LBTC rate for encounters with blood specimen collection performed by ED phlebotomy was found to be 15.8% (95% CI, 10.4%–23.1%), and 36.2% (95% CI, 31.0%–41.7%) for collections performed by ED nursing. Due to smaller sample sizes, larger LBTC rate variability was noted in the phlebotomy group as the DtR time increased.

## DISCUSSION

In addition to prolonged wait times, patient-specific and departmental factors have been shown to predict LBTC rates. Patients of younger age, male gender, a lower socioeconomic status group (including being uninsured or covered by Medicaid), non-English speaking or from a minority group, and with lower acuity presentations are at a higher risk of incomplete visits.^6^,^11^,^12^,^17^,^18^,^22^–^26^ Additionally, department specific-predictors include visits in a metropolitan or urban area, encounters at a teaching institution, and a lack of department management by an EM-trained physician.^12^,^15^,^22^,^23^ While ED providers have little control over patient-specific or institution-location predictors, at the departmental level changes can be implemented to identify and retain patients at higher risk for leaving early.

Initiatives aimed at reducing ED LBTC rates often target the “bottleneck” effect created during the patient arrival process.^12^ A variety of approaches directed at disrupting the arrival bottleneck, through increased operational efficiency and reduction in the time from patient arrival to provider evaluation, have been successfully demonstrated in the literature.^12^,^27^–^29^ Approaches include the implementation of an ED fast track for lower acuity patients, a “team” approach to patient triage including a physician-in-triage screening evaluation, the initiation of patient treatments during the triage process, and dedicated ED technicians performing minor procedures on waiting patients.^21^,^30^–^32^ Such approaches have demonstrated significant reduction in patient wait times, door to physician evaluation, and total LOS.^21^,^32^–^42^ Further, such approaches have been demonstrated to reduce LBTC rates.^21^,^32^,^36^,^38^,^39^,^42^,^43^,^46^ Unfortunately, deployment of extensive changes to the ED arrival process and triage system may not be feasible due to significant development time, effort, and expense.

To our knowledge, the impact of an ED phlebotomist encounter on premature departure rates has not been previously evaluated. In the study population, the ED phlebotomist group demonstrated a significant reduction in LBTC rates as compared to encounters when ED phlebotomy was not involved in the patient care process. This reduction in LBTC occurred despite an overall longer time from DtR, physician assessment, and disposition for encounters that included ED phlebotomy. Further, the impact on LBTC rates increased by half a percent for every 10-min increase in DtR, representing a population of encounters increasingly difficult to maintain in the ED. While the reason for the reduction in LBTC rates is unclear, it is reasonable to infer that patients who perceive their care has begun or is ongoing may be more inclined to wait longer to completion. Departments with limited ability to significantly change the patient arrival process may want to consider deployment of an ED phlebotomist in triage to reduce LBTC rates.

Dedicated ED phlebotomists offer additional advantages to patient care. Prior studies have demonstrated an increase in the rate of effective phlebotomy, improved patient satisfaction, a reduction in hemolysis, contamination, and specimen-misidentification rates. Further, phlebotomy utilization has been shown to result in a reduction in cost to the patient and hospital system, decreased needle-stick injury rates among providers, and a potential reduction in ED LOS.^44^–^50^ In addition to the reduction in ED LBTC rates, institutions should consider these additional advantages when considering the implementation of ED phlebotomists.

## LIMITATIONS

During study development and implementation, we identified several potential limitations. During the study window, a dedicated ED fast track was implemented, which altered patient flow. However, the lack of a formal ED fast track would likely have amplified study findings, as most ED fast track patients are of lower acuity, do not get blood drawn, and therefore are more likely to leave sooner.^12^ This study was conducted retrospectively in a single, adult ED that uses a provider-in-triage, fast track, and ED technicians. Generalizability to dissimilar departments may be limited.

While ESI (ESI 3) and patient age (42.03 vs. 42.29) were similar, additional patient demographics, including chief complaint, were not obtained and may limit comparison of the study groups. There was a predominance of female patients in the group that received ED phlebotomy collection. Previous studies have demonstrated a lower LBTC rate among female patients, which may have impacted the study results. Additionally, patients without blood specimen-collection orders after a physician-in-triage evaluation, by definition, were grouped with the patients without phlebotomy. This may have confounded the study results, as these patients were potentially lower risk, and as a result, more likely to LBTC. However, the distribution of patients by ESI in both study groups was similar.

Median DtR, primary physician encounter, and disposition was prolonged in the ED phlebotomy group. We believe that this was the result of ED crowding during the ED phlebotomist shift timeframe rather than a negative effect of ED phlebotomy. Patient disposition, including LBTC designation, is assigned by nursing providers in real time. Nursing is trained to assign the correct disposition designation, but it is possible that the incorrect disposition type may have been applied at times, as it was not possible to review each chart for confirmation. When not performing blood specimen collections, the ED phlebotomist was tasked with assisting with other department tasks, including stocking and performing electrocardiograms. While the impact of the performance of these tasks was not quantifiable as part of this study, it is possible that utilization of dedicated ED phlebotomists would also increase the impact on LBTC rates.

## CONCLUSION

The utilization of ED phlebotomy in waiting patients resulted in a significant reduction in ED LBTC rates. Further, as DtR times increased, the impact of ED phlebotomy became increasingly significant. Adult EDs with increased LBTC rates may want to consider the implementation of ED phlebotomy.

## Figures and Tables

**Table 1:** Patient demographic and throughput characteristics.

Demographics and throughput characteristics	Encounters with ED phlebotomist collection	Encounters without ED phlebotomist collection
Total encounters	1842	15507
Age (median)	42.03	42.29
Female (%)	1288 (69.92%)	8243 (53.16%)
Admissions (%)	91 (4.94%)	1158 (7.47%)
Emergency Severity Index (ESI) (Median)	3.0 (IQR 3–4)	3.0 (IQR 3–4)
Median door to (minutes):		
Triage	17.98	18.48
Room	122.71	74.12
Blood draw	66.16	152.26
Primary physician evaluation*	207.10	149.21
Disposition	343.76	286.76

*IQR*, interquartile range; *ED*, emergency department.

* Primary physician evaluation subsequent to physician-in-triage screening.
